# Pregnancy Outcomes of Different Endometrial Preparation in Patients With a History of Cesarean Section

**DOI:** 10.3389/fendo.2022.813791

**Published:** 2022-06-30

**Authors:** Run Xin Gan, Yuan Li, Juan Song, Quan Wen, Guang Xiu Lu, Ge Lin, Fei Gong

**Affiliations:** ^1^ Institute of Reproductive and Stem Cell Engineering, Central South University, Changsha, China; ^2^ Reproductive Medicine Center, Reproductive and Genetic Hospital of CITIC-XIANGYA, Changsha, China; ^3^ Key Laboratory of Stem Cells and Reproductive Engineering, National Health and Family Planning Commission, Changsha, China

**Keywords:** caesarean section, endometrial preparation, frozen embryo transfer, miscarriage, artificial reproductive technologies (ART)

## Abstract

**Objective:**

To investigate the efficacies of three cycle regimens in women receiving frozen embryo transfer with a history of cesarean section: natural cycle treatment, hormone replacement therapy and treatment with gonadotropin-releasing hormone agonist.

**Design:**

Retrospective cohort study.

**Methods:**

patients (N = 6,159) with a history of caesarean section who fulfilled the inclusion criteria were enrolled in the study from January 2014 to December 2019 at the CITIC-Xiangya Hospital of Reproduction and Genetics. Reproductive outcomes of patients in the natural cycle (n = 4,306) versus hormone replacement therapy (n = 1,007) versus gonadotropin-releasing hormone agonist + hormone replacement therapy groups (n = 846) were compared. Continuous data were analyzed using Student’s t-test, and categorical variables were analyzed using the χ2 test. Multivariable logistic regression was used to evaluate the possible relationships between the types of endometrial preparation and pregnancy outcomes after adjusting for confounding factors.

**Results:**

The unadjusted odds of the miscarriage rate of singleton pregnancies were significantly higher in the hormone replacement therapy compared with the natural cycle (25.5% versus 20.4%, respectively). After adjusting for possible confounding factors, the early miscarriage rate and the miscarriage rate of singleton pregnancies remained significantly higher in the hormone replacement therapy than the natural cycle. The clinical pregnancy rates in the natural cycle, hormone replacement therapy and gonadotropin- releasing hormone agonist + hormone replacement therapy of women with a history of cesarean section was 48.8%, 48% and 47.1%, respectively, and the live birth rates were 37%, 34.1% and 35.7%, respectively.

**Conclusions:**

In women undergoing frozen embryo transfer with a history of cesarean section, hormone replacement therapy for endometrial preparation was associated with a higher early miscarriage rate, albeit after statistical adjustment for confounding factors. However, the risk observed was little and did not influence the overall reproductive performances.

## Introduction

In 160 countries surveyed, 29.7 million (21.1%) live births were delivered *via* cesarean section (CS) in 2015, almost doubling the prevalence of CSs performed in 2000 (12.1%) (1). More than 30% of births in the USA and Australia (2, 3), and 40% to 50% in China and Brazil (4, 5) were delivered by CS. These trends are driven by older age, pregnancy complications, requests for CS, commercial reasons, litigation and assisted reproductive technology (ART) (6–8), which is associated with greater odds of CS delivery compared to the routine prenatal care of fertile women (6, 9).

CS is a risk factor for lower rates of fertility/infertility and early miscarriage, including ectopic pregnancy and spontaneous abortion (10). Lower pregnancy, implantation rates and live births after *in vitro* fertilization (IVF) or intracytoplasmic sperm injection (ICSI) (11–13)may be due to intracavitary fluid (ICF) from hormonal stimulation for controlled ovarian stimulation (COS) in patients with an isthmocele after a CS (14).

In 2015, the Chinese government introduced the second-child policy. Hence, many women with a history of a CS delivery conceived through ART are in need of frozen embryo transfer (FET) for their second progeny. Various protocols for endometrium preparation have been used to provide an optimal uterine environment for the transfer of thawed embryos, but the evidence supporting the superiority of one protocol over another is insufficient (15, 16). Therefore, the purpose of the present study was to compare the pregnancy outcomes following different FET protocols among women who have undergone CS.

## Materials and Methods

### Study Design and Participants

A retrospective study was conducted at the CITIC-Xiangya Hospital of Reproduction and Genetics. The Institutional Ethics Committee of the School of Basic Medical Science, Central South University approved the study’s protocol(2021-KT50).

Infertile women with a history of CS undergoing FET cycles were enrolled from January 2014 to December 2019. The exclusion criteria were: > 40 years of age at oocyte retrieval, history of multiple CSs and impaired cesarean scar healing, recurrent spontaneous abortion, recurrent implantation failure, preimplantation genetic testing, previous uterine myomectomy or operative hysteroscopy for intrauterine adhesions, thin endometrium (< 7 mm on the day of embryo transfer), untreated hydrosalpinx, adenomyosis, autoimmune or endocrine disease or missing records in the electronic database. Polycystic ovary syndrome (PCOS) were diagnosed by the Rotterdam diagnostic criteria and endometriosis by laparoscopy or pathology. Based on their endometrial protocols, the women included in the study were divided into three groups: (i) natural cycle (NC), (ii) hormone replacement therapy (HRT) and (iii) gonadotropin-releasing hormone agonist (GnRH-a) + HRT-groups.

### Endometrial Preparation Before Embryo Transfer

The decision to proceed with the NC-, HRT- or GnRH-a-group was determined by physician guidance and patient preference. In our clinical practice, for patients of PCOS without an increased LH/FSH ratio, we choose hormone replacement therapy or natural cycle, otherwise we use GnRH-a in combination with hormone replacement therapy. If a patient has moderate-to-severe endometriosis, we choose GnRh-a combined with hormone replacement therapy for them. And natural cycle was chosen for patients due to male factor infertility, tubal factor infertility or unexplained infertility. All women were screened for endometrial thickness using transvaginal sonography, and blood samples were taken to measure luteinizing hormone (LH), estradiol and progesterone levels before performing the FET. And before embryo transfer, endometrial lesins such as endometrial polyps, submucous myomas and chronic endometritis would be managed by hysteroscopy, antibiotic treatment. If the endometrial thickness was < 8 mm on the day of embryo transfer in any protocols, the cycle was cancelled.

Participants in the NC-group did not take any medication or throughout the follicular phase, which has been similarly described in work published by our group (17). After ovulation, the luteal phase was supported with dydrogesterone (600 mg/day, 3 × 200 mg; Duphaston, Abbott Biologicals B.V., The Netherlands).

Participants in the HRT-group began taking oral estradiol valerate (Progynova, Delpharm Lille SAS, France) on the third day of a natural or progesterone-induced menstrual cycle. The drug was administered either as a fixed dose (6 mg daily) or an incremental dose (2 to 6 mg daily). A vaginal ultrasound examination was conducted 10–15 days later to measure endometrial thickness and ensure that no dominant follicle emerged. If the endometrial thickness was < 8 mm, the estrogen dosage was increased to 8 mg/d for another week. When endometrial thickness reached 8 mm, dydrogesterone (10 mg per 12 h; Duphaston, Abbott Biologicals BV, The Netherlands) and the progesterone medication, Utrogestan (200 mg, three times a day; Laboratoires Besins International, France) were administered orally and vaginally, respectively, to provide luteal phase support until 10 weeks of gestation if a pregnancy had occurred. Embryo transfer (ET) was performed three days after dydrogesterone and progesterone were administered for the day-3 embryos or five days later for blastocysts.

Participants in the GnRH-a + HRT-group received a depot injection of long-acting GnRH-a Triptorelin (1.875 mg, Ferring GmbH, Kiel, Germany). Twenty-one days after receiving the GnRH-a injection, the women underwent ultrasound examinations and blood tests to measure their levels of serum follicle stimulating hormone, luteinizing hormone and estradiol to confirm complete pituitary downregulation before beginning exogenous hormone supplementation, which commenced on the third day without bleeding with a referral to HRT-FET.

### Embryo Vitrification, Thawing and Transfer

Embryos were vitrified using the Kitazato Embryo Vitrification Kit (Kitazato Biopharma Co, Ltd) using high-security vitrification straws (Cryo Bio System). The embryos were transferred to a commercially available warming solution for thawing (Kitazato Biopharma), following the manufacturer’s instructions.

Cleavage-stage embryos (day 3) were graded according to the appearance of the blastomeres and the percentage of fragments, using conventional criteria (18). Cleavage embryos were considered high quality if they met the following criteria: i) their fertilization was normal; ii) they had at least six blastomeres, iii) the blastomere size was stage-specific, iv) the percentage of embryo fragments did not exceed 10%; v) the blastomere was transparent and without cytoplasmic inclusions or vacuoles; and vi) there were no multinucleated blastomeres. Suboptimal day 3 embryos were placed in a culture for an extended period to develop to the blastocyst stage. The blastocyst quality assessment was based on the scoring system of Gardner and Schoolcraft (19), with embryos graded ≥ 3 BB considered to be good blastocysts. No more than two embryos were transferred in the FET cycles. Physicians usually recommend blastocyst culture and transfer for patients with a history of CS. However, the final embryo stage selection depends on the patients’ decision.

### Outcome Parameters and Statistical Methods

The study’s primary outcome was the live birth rate per ET. The secondary endpoints included the clinical pregnancy, miscarriage, implantation and heterotopic pregnancy rates. A live birth was defined as a live born baby after 24 gestational weeks. A clinical pregnancy was defined as the presence of at least one intrauterine gestation sac on ultrasound at 28 days after the ET. Miscarriage was defined as a pregnancy loss before the 24th gestational week, whereas early miscarriage was defined as a pregnancy loss before the 12th gestational week.

Continuous data were analyzed using Student’s *t*-test, and categorical variables were analyzed using the χ^2^ test. Multivariable logistic regression was used to evaluate the possible relationships between the types of endometrial preparation and pregnancy outcomes after adjusting for confounding factors. including age at ET, BMI, infertility duration, cause of infertility, duration of cryopreservation, comorbidities, uterine malformation, serum progesterone level on the day before transplantation, endometrial thickness on the ET day, high-quality embryo transfer, number of embryos transferred and stage of embryo development. All statistical analyses were performed using SPSS version 21.0. A *P-*value <.05 was considered statistically significant.

## Results

### Study Population

Data from the 6,159 patients who fulfilled the inclusion criteria were analyzed, with no loss to follow-up. Among them, 4,306 women underwent NC treatment, 1,007 received HRT and 846 received GnRH-a + HRT.

### Baseline Characteristics

Patients’ baseline characteristics are presented in **Table 1**. No significant differences in age of oocyte retrieval or age at ET among the three treatment groups were observed. Due to the study’s large sample size, smaller differences between the participants at baseline may have led to statistically significant group differences in body mass index, infertility duration, cause of infertility and duration of cryopreservation. The proportions of polycystic ovary syndrome (PCOS) and endometriosis (EMS) that contributed to infertility in women who received ART were significantly higher in the GnRH-a + HRT-group than the NC- or HRT-group. The percentage of complications caused by uterine malformation was not statistically significant.

**Table 1:** Baseline characteristics of participants in the three cycles.

Characteristic		NC (n = 4306)	HRT (n = 1007)	GnRH-a+HRT (n = 846)	*P*
Age at embryo transfer (y)		34.0 (31.0, 37.0)	34.0 (31.0, 37.0)	34.0 (31.0, 37.0)	0.954
Age at oocyte retrieval (y)		32.2 (28.8, 35.8)	32.8 (28.8, 35.9)	32.5 (29.0, 35.8)	0.052
BMI (kg/m^2^)		21.9 (20.4, 23.6)	22.2 (20.8, 23.6)*	22.0 (20.4, 23.8)	0.004
Infertility duration (years)		3 (2, 5)	4 (2, 6)*	4 (3, 6)*	<0.001
Cause of infertility					
	Male factors	720/4306 (16.7%)	119/1007 (11.8%)*	96/846 (11.3%)*	<0.001
	Tubal factors	3611/4306 (83.9%)	904/1007 (89.8%)*	756/846 (89.4%)*	<0.001
	PCOS+ ovulatory dysfunction	226/4306 (5.25%)	140/1007 (13.9%)*	159/846 (18.8%)*#	<0.001
	Endometriosis	142/4306 (3.30%)	34/1007 (3.38%)	109/846*# (12.9%)	<0.001
Uterine malformation		165/4306 (3.83%)	45/1007 (4.47%)	43/846 (5.08%)	0.201
Duration of cryopreservation (y)		0.5 (0.2, 3.3)	0.3 (0.2, 2.5)*	0.5 (0.3, 2.8)#	<0.001

*Compared to the NC, P < 0.05; # = compared to the HRT cycle, P < 0.05; NC, natural cycle; HRT, hormone replacement therapy; GnRH-a, gonadotropin-releasing hormone agonist; PCOS, polycystic ovary syndrome.

### Cycle Characteristics of the FET

As presented in **Table 2**, the proportion of the day 3 embryos that were transferred was significantly lower in the HRT-group than the NC-group, while the proportion of the day 5 embryos that were transferred was significantly higher in the HRT-group than the NC-group. The distribution of the best embryos transferred was significantly higher in the NC-group than the HRT-group. Endometrial thickness on the day of ET was significantly greater in the NC-group than the other two groups. Serum progesterone levels on the day before transplantation were comparable among the three study groups. The embryo survival rate after thawing and the number of embryos transferred were also similar across the study groups.

**Table 2:** Cycle characteristics during the embryo transfer.

Characteristic		NC (n = 4306)	HRT (n = 1007)	GnRH-a+HRT (n = 846)	*P*
Serum progesterone levels on the day before transplantation (ng/ ml)		9 (6.4, 13.1)	9.3 (7, 12.4)	9 (6.7, 11.9)	0.257
Embryo stage at transfer, n (%)					0.007
	Cleavage (%)	996/4306 (23.1%)	191/1007 (19.0%)*	190/846 (22.5%)	
	Blastocyst (%)	3237/4306 (75.2%)	797/1007 (79.1%)*	631/846 (74.6%)	
	Cleavage +Blastocyst (%)	73/4306 (1.70%)	19/1007 (1.89%)	25/846 (2.96%)*	
Number of embryos transferred					0.154
	1	2561/4306 (59.5%)	614/1007 (61.0%)	479/846 (56.6%)	
	2	1745/4306 (40.5%)	393/1007 (39.0%)	367/846 (43.4%)	
Post-thaw embryo survival rate		14452/14936 (96.8%)	3279/3385 (96.9%)	3098/3219 (96.2%)	0.274
High quality embryo transfer (%)		2249/4306 (52.2%)	482/1007 (47.9%)*	444/846 (52.5%)	0.038
Endometrial thickness (mm) on the day of ET		11.9 (10.7, 13.2)	11.5 (10.6, 12.5)*	11.3 (10.4, 12.4)*	<0.001

*Compared to the NC, P < 0.05; # = compared to the HRT cycle, P < 0.05; NC, natural cycle; HRT, hormone replacement therapy; GnRH-a, gonadotropin-releasing hormone agonist; PCOS, polycystic ovary syndrome; ET, embryo transfer.

### Reproductive Outcomes

Reproductive outcomes are shown in **Table 3**. The live birth rates per ET were comparable in the three groups. The early miscarriage rate and miscarriage rate of singleton pregnancies were significantly higher in the HRT-group compared with the NC-group. The rates of clinical pregnancy, implantation and heterotopic pregnancy were similar among the three groups. The rates of miscarriage did not differ significantly by group, but the early miscarriage rate was somewhat lower in the GnRH-a+ HRT-group.

**Table 3:** Reproductive outcomes per embryo transfer.

Characteristic		NC	HRT	GnRH-a+HRT (n = 846)	*P*
		(n = 4306)	(n = 1007)		
Clinical pregnancy rate		2103/4306 (48.8%)	483/1007 (48.0%)	399/846 (47.1%)	0.633
Implantation rate		2464/6051 (40.7%)	549/1400 (39.2%)	461/1213 (38.0%)	0.162
Heterotopic		28/2103	10/483	5/399	0.444
pregnancy		-1.33%	-2.30%	-1.25%
Twins and multiple pregnancies		312/4306 (7.25%)	52/1007	33/846*	<0.001
			-5.16%	-3.90%
Miscarriage rates(1st or 2nd trimester)		481/2103 (22.9%)	132/483 (27.3%)	90/399	0.101
				-22.60%
	1st	394/2103 (18.7%)	117/483 (24.2%)*	74/399	0.021
	trimester			-18.50%
	2nd trimester	87/2103	15/483	16/399	0.576
		-4.14%	-3.11%	-4.01%
	Miscarriage rate of singleton pregnancies	428/2103 (20.4%)	123/483 (25.5%)*	79/399	0.036
				-19.80%
	Miscarriage rate of multiple pregnancies	53/2103	9/483	11/399	0.632
		-2.52%	-1.86%	-2.76%
Stillbirths		2/2103 (0.0951%)	0	1/399	0.5
				-0.25%
Live birth rate		1595/4306 (37.0%)	343/1007 (34.1%)	302/846 (35.7%)	0.19
Singletons		1402/4306 (32.6%)	307/1007 (30.5%)	269/846	0.437
				-31.80%
Twins		193/4306 (4.48%)	36/1007 (3.57%)	33/846	0.377
			-3.90%
Preterm births		214/2103 (10.2%)	57/483	36/399	0.383
		-11.80%	-9.02%

*Compared to the NC, *P* < 0.05; # = compared to the HRT cycle, *P* < 0.05; NC, natural cycle; HRT, hormone replacement therapy; GnRH-a, gonadotropin-releasing hormone agonist.

After adjustment for the above-mentioned confounding factors (**Table 4**), the early miscarriage rate and miscarriage rate of singleton pregnancies remained higher in the HRT-group compared to the NC-group.

**Table 4:** Unadjusted and adjusted ORs of the reproductive outcomes following the NC versus HRT and GnRH-a+HRT.

Reproductive outcomes	Unadjusted OR (95%CI)		Adjusted OR (95%CI)	
	HRT	GnRH-a+HRT	HRT	GnRH-a+HRT
Clinical pregnancy rate	0.966	0.935	0.982	0.929
	(0.842, 1.108)	(0.807, 1.084)	(0.851, 1.134)	(0.793, 1.088)
Heterotopic pregnancy	1.532	0.908	1.633	0.855
	(0.742, 3.165)	(0.35, 2.359)	(0.771, 3.461)	(0.314, 2.325)
Miscarriage rate (1st trimester 2nd trimester)	1.2	0.947	1.208	0.933
	(0.976, 1.474)	(0.746, 1.201)	(0.978, 1.492)	(0.727, 1.196)
Early miscarriage rate	1.305	0.952	1.339	0.97
	(1.049, 1.625)	(0.734, 1.234)	(1.07, 1.675)	(0.739, 1.272)
Miscarriage rate of singleton births	1.261	0.933	1.266	0.928
	(1.018, 1.561)	(0.725, 1.201)	(1.017, 1.575)	(0.713, 1.208)
Live birth rate	0.878	0.944	0.883	0.941
	(0.76, 1.014)	(0.809, 1.1)	(0.76, 1.027)	(0.798, 1.109)

With NC as the reference group; OR, odds ratio; CI, confidence interval; NC, natural cycle; HRT, hormone replacement therapy; GnRH-a, gonadotropin-releasing hormone agonist.

## Discussion

The rate of early miscarriage of pregnancy per embryo transfer (ET) was higher in the HRT-group than the natural cycle (NC) group (24.2% versus 18.7%, respectively). The unadjusted odds of the miscarriage rate of singleton pregnancies were also significantly higher in the HRT-group compared with the NC-group (25.5% versus 20.4%, respectively). After adjusting for possible confounding factors, the early miscarriage rate and the miscarriage rate of singleton pregnancies remained significantly higher in the HRT-group than the NC-group. The clinical pregnancy rates in the NC-, HRT- and GnRH-a + HRT-groups of women with a history of CS was 48.8%, 48% and 47.1%, respectively; the heterotopic pregnancy rate was 1.33%, 2.30% and 1.25%, respectively, and the live birth rates were 37%, 34.1% and 35.7%, respectively. These findings imply that HRT may associated with increased the risk of early miscarriage during FET cycles in patients with a history of CS.

Our results are consistent with those of a retrospective cohort study that found increased early miscarriage after FET in women with previous CS (20). These results were supported by Naji’s research (21). These studies suggest a positive association between a scarred uterus and a higher spontaneous miscarriage rate. There is increasing evidence that a large number of early miscarriages are caused by impaired decidualization (22). It is possible that the presence of CS scarring further aggravates this process. Two studies reported that a history of CS leads to a defect in the anterior lower segment of the uterus in 42%-58% of women (23, 24). Another study found fewer leukocytes and less vascularization at the scar site than in the endometrium of women with an unscarred uterus (25). A delay in endometrial maturation, which was also found at the scar site, was caused by disruption in steroid receptor expression. However, this total was obtained by summing the results of all the endothelial preparation protocols.

Based on previous researches, we also investigate the efficacies of three cycle regimens in women receiving frozen embryo transfer (FET) with a history of CS, which found that hormone replacement therapy for endometrial preparation further associated with increased the risk of early miscarriage in patients with history of CS. However, the risk observed was little and did not influence the overall reproductive performances. This may be due to the small sample sizes in the cohorts. Embryos are transferred to the endometrium prepared by either normal ovulation or hormonal replacement with or without a gonadotrophin releasing hormone agonist. Another research suggests high miscarriage rates are related to PCOS, high and low body mass index (26), an environment with excessive estrogen or a suboptimal ratio between progesterone and estradiol (27). However, the proportion of PCOS was higher in the HRT + GnRh-a-group than the other two groups in the present study. Although there was no statistical difference in pregnancy outcome between HRT and GnRH-a+HRT, the miscarriage rate was lower in GnRh-a+HRT, which was similar to NC, than in HRT. Therefore, for patients in CS with PCOS patients for FET, GnRH-a + HRT may be a more suitable choice. Moreover, our subgroup analysis (**Supplementary Tables S1–S3**) based on infertility factors showed that pregnancy outcomes were better in the GnRH-a group than in the other groups for PCOS, although there was no statistical difference. Meanwhile, for the PCOS patients, some studies have shown that after adjusting for possible confounding factors, the rates of pregnancy loss remained consistently lower in the letrozole-stimulated FET group than in the HRT-FET group (28). However, further studies and randomized controlled trials are required to determine whether letrozole-stimulate cycle is beneficial in patients with PCOS combined with history of CS.

The regression analysis in the present study that adjusted for important confounders revealed the risk of early miscarriage was significantly higher in women with a history of CS using the HRT protocol than in the women using the NC protocol. Thus, age at ET, BMI, infertility duration, cause of infertility, duration of cryopreservation, comorbidities, uterine malformation, serum progesterone level on the day before transplantation, endometrial thickness on the ET day, high-quality embryo transfer, number of embryos transferred and stage of embryo development did not play an independent role in early miscarriage after FET of women with history of CS. According to some studies, in general population, early miscarriage rates are higher with HRT-endometrial preparation for FET than other protocols, even though the pregnancy rates were similar (15, 29, 30). And our subgroup analysis based on infertility factors,which are in line with findings of several these studies, found that for patients with tubal factors, early miscarriage rates and singleton miscarriage rates were significantly higher in the HRT group than in the other endo-preparation regimen groups, even after adjusting for confounding factors (**Supplementary Tables S4–S7**). The randomized prospective study of Cerrillo et al. led in 2011–2012 on 570 FET cycles found a first-trimester early pregnancy loss rate of 21.2% with AC, 12.9% with NC and 11.1% with modified NC (P = 0.01) (31). These rates are lower than those reported in our study but inclusion and exclusion criteria differed: patients were under 39 years of age and had regular cycles, and patients with stage III/IV endometriosis and polycystic ovary syndrome were excluded. In a retrospective analysis of 1132 FET, Veleva et al. (2008) showed that the early pregnancy loss rate was 1.7 times higher in AC than in the NC and fresh embryo transfer cycles (26).

Overall, the higher rates of early pregnancy loss reported with AC might be explained by defective placentation. Moreover, the one of complications of CS is placental abnormalities. Therefore, these may be a possible reason for the high early miscarriage rate after FET with HRT in patients with a history of CS. In addition, a real or relative deficiency in progesterone during early pregnancy might increase the risk of early pregnancy loss. However, no clear guideline concerning a progesterone level cut-off exists so far. In our study, luteal phase support by progesterone was homogeneous overall. Altogether, the exact mechanisms underlying the enhanced rates of early pregnancy losses reported in our study remain to be established. As we all know, embryo aneuploidy has been validated as one of the most important factors for miscarriage. However, information on the causes of miscarriage is incomplete. Further studies need to consider the population with a history of CS in preimplantation genetic testing cycle. Given the high risk of CS-related miscarriages, we hypothesize that exogenous estrogen and progesterone could not improve the endometrial environment and that decidualization was impaired and steroid receptor expression disrupted by the presence of a CS scar resulting in later implantation within the window or an implantation site closed to the scar. Future research should explore the relationship and mechanisms that steroid hormones and increased early miscarriage in patient with a history of CS. Altogether, the exact mechanisms underlying the enhanced rates of early pregnancy losses reported in our study remain to be established.

Interestingly, our study also found that the clinical pregnancy and live birth rates in the HRT + GnRh-a-group were similar to the rates of the other two groups, whereas the miscarriage and early miscarriage rates were lower than the other two groups, although not significantly lower. This finding may be related to the role of GnRH. Research has found GnRH expression in the endometrium can directly inhibit inflammatory factors and increase endometrial adhesion molecules (32, 33). Indeed, two retrospective studies (34, 35) have reported benefits of GnRH-a pretreatment on pregnancy outcomes following artificial-cycle frozen-thawed embryo transfers, including improved clinical pregnancy rates and lower pregnancy loss rates.

The short- and long-term complications of CS are infection, increased hemorrhage risk, reduced fertility and increased risk of obstetric complications in subsequent pregnancies (placental abnormalities, caesarean scar pregnancies and uterine rupture) (36, 37). Given these risks, studies have examined the impact of previous CS on infertility and reproductive outcomes (rates of reduced pregnancy, live births, transplantation and early miscarriage) in COS cycles (11–13). However, even in the “in phase” endometrium, the supraphysiological steroid levels achieved with COS may negatively affect endometrial receptivity (38, 39). The risk of developing intracavitary fluid during hormonal stimulation for IVF was almost 40% in patients with an existing isthmocele after a previous CS delivery (14). Theoretically, the accumulation of fluid and mucus may facilitate bacterial growth, reducing the chances of successful IVF (40). To some extent, FET provides better endometrial receptivity by avoidance of supraphysiological steroid levels and adverse effects of COS (41, 42). Many women with a history of CS by ART have been in need of FET for their second progeny since the release of the second-child policy. Given the importance of endometrial preparation for FET success, physicians should improve their understanding of the effects of different protocols on pregnancy outcomes in patients with a CS history.

According to our study, HRT is associate with an increased risk of early miscarriage during FET cycles in patients with a history of CS. Physicians should carefully consider using HRT in patients of CS.

The study provides new insights into current patterns of practice and associated clinical outcomes; moreover, it is the first to investigate the efficacy of different endometrial preparation protocols used for FETs in patients with a CS history. The limitations of this study include its retrospective design, such as selection bias about endometrial preparation protocol, confounding factors about proportion of cause of infertility. In clinical practice, HRT protocol for PCOS patients, GnRH-a+HRT protocol for endometriosis are common in many IVF centers. Based on our available studies, it is recommended to consider the use of GnRH-a-HRT regimen in patients of CS with PCOS thus reducing the miscarriage rate, although there is no statistical difference. However, further studies and randomized controlled trials in subgroups are necessary to clarify the impact of the regimen on pregnancy outcomes in patients with a history of CS. Furthermore, the detailed ultrasound information on uterine myometrial defects, complications related to CS like isthmocele and intracavitary fluid were unavailable in our study. Another limitation is that pregnancy-related complications and neonatal outcomes were not analyzed, as this information was collected during a telephone follow up and could not be verified for analysis. Further studies and randomized controlled trials are required to document the complications of CS and compare the maternal and neonatal safety of the protocols examined in this study.

## Conclusion

In women undergoing FET with a history of CS, HRT for endometrial preparation was associated with a higher early miscarriage rate. However, the risk observed was little and did not influence the overall reproductive performances.

## Data Availability Statement

The raw data supporting the conclusions of this article will be made available by the authors, without undue reservation.

## Ethics Statement

The studies involving human participants were reviewed and approved by School of Basic Medical Science, Central South University. The patients/participants provided their written informed consent to participate in this study.

## Author Contributions

Conceived and designed the experiments: FG, GL and GXL Performed the experiments: RG Organized the data: YL and JS Contributed reagents/materials/analysis tools: QW Wrote the manuscript: RG. All authors contributed to the article and approved the submitted version.

## Funding

We are grateful to the financial support received the National Science Foundation of China (81501328) for the decision to submit the article for publication.

## Conflict of Interest

The authors declare that the research was conducted in the absence of any commercial or financial relationships that could be construed as a potential conflict of interest.

## Publisher’s Note

All claims expressed in this article are solely those of the authors and do not necessarily represent those of their affiliated organizations, or those of the publisher, the editors and the reviewers. Any product that may be evaluated in this article, or claim that may be made by its manufacturer, is not guaranteed or endorsed by the publisher.
