# Supplementary material for: Pregnancy Outcomes of Different Endometrial Preparation in Patients With a History of Cesarean Section
Source: Front Endocrinol (Lausanne). 2022 Jun 30;13:813791. doi: 10.3389/fendo.2022.813791 (PMC9280671; doi:10.3389/fendo.2022.813791)
Supplement: Supplementary file 1 [file DataSheet_1.docx]

Supplemental materials

Subgroups of PCOS

Table S1. Basic characteristics at cycle level.

| Characteristic | | NC  （n=226） | HRT  （n=140） | GnRH-a+HRT（n=159） | P |
| --- | --- | --- | --- | --- | --- |
| Age at embryo transfer(y) |  | 32.0  （29.0，35.0） | 32.0  （29.0，35.0） | 32.0  （30.0，35.0） | .897 |
| Age at oocyte retrieval(y) |  | 29.6  （27.3，33.0） | 29.8  （27.0，33.1） | 29.8  （27.8，32.6） | 0.959 |
| BMI (kg/㎡) |  | 22.3  （20.6，24.1） | 22.6  （21.2，24.6） | 22.3  （20.8，24.2） | 0.142 |
| infertility duration(years) |  | 3.0  （2.0，4.0） | 4.0  （2.0，6.0）* | 4.0  （3.0，6.0）* | 0.002 |
| duration of cryopreservation(y) |  | 0.8（0.3，3.4） | 0.5（0.3，3.3） | 0.7（0.3，3.6） | 0.231 |

*：compared to NC，p<0.05； #：compared to HRT，p<0.05

Table S2. Cycle characteristics at transfer level.

| Characteristic | | NC  （n=226） | HRT  （n=140） | GnRH-a+HRT（n=159） | P |
| --- | --- | --- | --- | --- | --- |
| Serum progesterone levels on the day before transplantation(ng/ml) |  | 9.4（7.0，13.2） | 8.9（6.2，11.4） | 8.9（6.5，11.2） | 0.114 |
| Embryo stage at transfer, n (%) |  |  |  |  | 0.114 |
|  | Cleavage（%） | 33/226（14.6%） | 12/140（8.6%） | 28/159（17.6%） |  |
|  | Blastocyst（%） | 190/226（84.1%） | 124/14（88.6%） | 130/159（81.8%） |  |
|  | Cleavage +Blastocyst（%） | 3/226（1.3%） | 4/140（2.9%） | 1/159（0.6%） |  |
| Number of embryos transferred）（%） |  |  |  |  | 0.115 |
|  | 1 | 138/226（61.1%） | 98/140（70.0%） | 94/159（59.1%） |  |
|  | 2 | 88/226（38.9%） | 42/140（30.0%） | 65/159（40.9%） |  |
| Post-thaw embryo survival rate |  | 774/798（97.0%） | 517/52（98.1%） | 660/687（96.1%） | 0.123 |
| High quality embryo transfer (%) |  | 117/226（51.8%） | 64/140（45.7%） | 86/159（54.1%） | 0.329 |
| Endometrium thickness on the day of ET (mm)) |  | 12.0  （10.7，13.3） | 11.4  （10.7，12.7） | 11.4  （10.5，12.4）* | 0.002 |

*：compared to NC，p<0.05； #：compared to HRT，p<0.05

Table S3. Reproductive outcomes per embryo transfer.

| Characteristic | | NC  （n=226） | HRT  （n=140） | GnRH-a+HRT（n=159） | p |
| --- | --- | --- | --- | --- | --- |
| Clinical pregnancy rate |  | 121/226（53.5%） | 77/140（55.0%） | 95/159（59.7%） | 0.470 |
| Implantation rate |  | 139/314  （44.3%） | 88/182  （48.4%） | 112/224（50.0%） | 0.390 |
| [heterotopic](#/javascript:;) [pregnancy](#/javascript:;) |  | 1/121  （0.83%） | 1/77  （1.30%） | 1/95  （1.05%） | 0.949 |
| Twins&multiple pregnancies |  | 11/226  （4.87%） | 10/140  （7.14%） | 15/159  （9.43%） | 0.215 |
| Miscarriage rate（1st trimester 2nd trimester） |  | 23/121（19.0%） | 16/77（20.8%） | 13/95（13.7%） | 0.429 |
|  | 1st trimester | 18/121（14.9%） | 15/77（19.5%） | 9/95（9.47%） | 0.172 |
|  | 2nd trimester | 5/121  （4.13%） | 1/77  （1.30%） | 4/95  （4.21%） | 0.492 |
|  | Miscarriage rate of singleton pregnancies | 22/121（18.2%） | 15/77（19.5%） | 11/95（11.6%） | 0.297 |
|  | Miscarriage rate of multiple pregnancies | 1/121  （0.83%） | 1/483  （0.21%） | 2/95  （2.11%） | 0.723 |
| Stillbirths |  | 0 | 0 | 0 |  |
| Live birth rate |  | 97/226（42.9%） | 60/140（42.9%） | 81/159（50.9%） | 0.235 |
| Singletons |  | 86/226（38.1%） | 50/140（35.7%） | 66/159（41.5%） | 0.581 |
| Twins |  | 11/226（4.9%） | 10/140（7.1%） | 15/159（9.4%） | 0.215 |
| Preterm birth |  | 12/226（5.3%） | 13/140（9.3%） | 13/159（8.2%） | 0.311 |

*：compared to NC，p<0.05； #：compared to HRT，p<0.05
