# Supplementary material for: Pregnancy Outcomes of Different Endometrial Preparation in Patients With a History of Cesarean Section
Source: Front Endocrinol (Lausanne). 2022 Jun 30;13:813791. doi: 10.3389/fendo.2022.813791 (PMC9280671; doi:10.3389/fendo.2022.813791)
Supplement: Supplementary file 2 [file DataSheet_2.docx]

Subgroups of tubal factors infertility

Table S4. Basic characteristics at cycle level.

| Characteristic | | NC  （n=3611） | HRT  （n=904） | GnRH-a+HRT（n=756） | P |
| --- | --- | --- | --- | --- | --- |
| Age at embryo transfer(y) |  | 34.0  （31.0，37.0） | 34.0  （31.0，37.0） | 34.0  （31.0，37.0） | 0.997 |
| Age at oocyte retrieval(y) |  | 32.6  （28.8，35.8） | 32.8  （29.3，36.2） | 32.7  （29.1，35.8） | 0.155 |
| BMI (kg/㎡) |  | 20.4  （20.4，23.6） | 22.2  （20.7，23.8）* | 22.0  （20.4，23.8） | 0.008 |
| infertility duration(years) |  | 3.0（2.0，5.0） | 4.0（2.0，6.0）* | 4.0（3.0，6.0）* | 0.000 |
| duration of cryopreservation(y) |  | 0.4（0.2，3.1） | 0.3（0.2，1.3）* | 0.4（0.3，2.8）*# | 0.000 |

*：compared to NC，p<0.05； #：compared to HRT，p<0.05

Table S5. Cycle characteristics at transfer level.

| Characteristic | | NC  （n=3611） | HRT  （n=904） | GnRH-a+HRT（n=756） | P |
| --- | --- | --- | --- | --- | --- |
| Serum progesterone levels on the day before transplantation(ng/ml) |  | 9.0（6.4，13.1） | 9.3（7.0，12.4） | 9.0（6.8，11.9） | 0.272 |
| Embryo stage at transfer, n (%) |  |  |  |  | 0.009 |
|  | Cleavage（%） | 855/3611（23.7%） | 174/904（19.2%）* | 177/756  （23.4%）# |  |
|  | Blastocyst（%） | 2691/3611（74.5%） | 714/904（79.0%）* | 556/756  （73.5%）# |  |
|  | Cleavage +Blastocyst（%） | 65/3611（1.8%） | 16/904（1.8%） | 23/756（3.0%） |  |
| Number of embryos transferred）（%） |  |  |  |  | 0.071 |
|  | 1 | 2111/3611（58.5%） | 546/904（60.4%） | 415/756  （54.9%） |  |
|  | 2 | 1500/3611  （41.5%） | 358/904（39.6%） | 341/756  （45.1%） |  |
| Post-thaw embryo survival rate |  | 102113/102496  （99.6%） | 2881/2974（96.9%）* | 2774/2889（96.0%）* | 0.000 |
| High quality embryo transfer (%) |  | 1926/3611（53.3%） | 434/904（48.0%）* | 396/756  （52.4%） | 0.016 |
| Endometrium thickness on the day of ET (mm)) |  | 11.9  （10.6，13.1） | 11.5  （10.5，12.5）* | 11.3  （10.4，12.3）* | 0.000 |

*：compared to NC，p<0.05； #：compared to HRT，p<0.05

Table S6. Reproductive outcomes per embryo transfer.

| Characteristic | | NC  （n=3611） | HRT  （n=904） | GnRH-a+HRT（n=756） | p |
| --- | --- | --- | --- | --- | --- |
| Clinical pregnancy rate |  | 1753/3611（48.5%） | 431/904（47.7%） | 348/756（46.0%） | 0.440 |
| Implantation rate |  | 2013/5111  （39.4%） | 479/1262  （38.0%） | 396/1097（36.1%） | 0.109 |
| [heterotopic](#/javascript:;) [pregnancy](#/javascript:;) |  | 27/1753  （1.5%） | 10/431  （2.30%） | 3/348  （0.9%） | 0.265 |
| Twins&multiple pregnancies |  | 158/3611  （4.4%） | 32/904  （3.5%） | 32/756  （4.2%） | 0.535 |
| Miscarriage rate（1st trimester 2nd trimester） |  | 412/1753（23.5%） | 121/431（28.1%） | 80/348  （23.0%） | 0.118 |
|  | 1st trimester | 335/1753（19.1%） | 107/431（24.8%）* | 64/348  （18.4%） | 0.021 |
|  | 2nd trimester | 77/1753  （4.4%） | 14/431  （3.2%） | 16/348  （4.6%） | 0.533 |
|  | Miscarriage rate of singleton pregnancies | 364/1753（20.8%） | 113/431（26.2%）* | 70/348  （20.1%） | 0.037 |
|  | Miscarriage rate of multiple pregnancies | 48/1753  （2.7%） | 8/431  （1.9%） | 10/348  （2.9%） | 0.556 |
| Stillbirths |  | 2/1753(0.11%) | 0 | 1/348(0.29%) | 0.419 |
| Live birth rate |  | 1315/3611（36.4%） | 302/904（33.4%） | 264/756（34.9%） | 0.215 |
| Singletons |  | 1157/3611（32.0%） | 270/904（29.9%） | 232/756  （30.7%） | 0.399 |
| Twins |  | 158/3611（4.4%） | 32/904（3.5%） | 32/756（4.2%） | 0.535 |
| Preterm birth |  | 178/175（10.2%） | 50/904（11.6%） | 31/756（8.9%） | 0.459 |

*：compared to NC，p<0.05； #：compared to HRT，p<0.05

Table S7 Unadjusted and adjusted odds ratios (ORs) of miscarriage rate following L-FET versus AC-FET.

|  | Unadjusted OR(95%CI) | | Adjusted OR(95%CI) | |  |
| --- | --- | --- | --- | --- | --- |
|  | HRT | GnRH-a+HRT | HRT | GnRH-a+HRT |  |
| Miscarriage rate（1st trimester 2nd trimester） | 1.270（1.002，1.611） | 0.972（0.739，1.277） | 1.293（1.015，1.647） | 1.006（0.761，1.329） |  |
| early miscarriage rate | 1.398（1.090，1.793） | 0.954（0.709，1.283） | 1.451（1.125，1.872） | 0.999（0.738，1.352） |  |
| miscarriage rate of singleton | 1.356（1.063，1.30） | 0.961（0.722，1.279） | 1.395（1.087，1.790） | 1.023（0.763，1.371） |  |

Note: Analyses were adjusted for confounding factors, including age at embryo transfer, body mass index, infertility duration, duration of cryopreservation, serum progesterone levels on the day before transplantation , endometrium thickness on the ET day, high quality embryo transfer, number of embryos transferred, and embryo developmental stage.
